# Expression of Heat Shock and Other Stress Response Proteins in Ticks and Cultured Tick Cells in Response to *Anaplasma* spp. Infection and Heat Shock

**DOI:** 10.1155/2010/657261

**Published:** 2010-09-29

**Authors:** Margarita Villar, Nieves Ayllón, Ann T. Busby, Ruth C. Galindo, Edmour F. Blouin, Katherine M. Kocan, Elena Bonzón-Kulichenko, Zorica Zivkovic, Consuelo Almazán, Alessandra Torina, Jesús Vázquez, José de la Fuente

**Affiliations:** ^1^Instituto de Investigación en Recursos Cinegéticos IREC (CSIC-UCLM-JCCM), Ronda de Toledo s/n, 13005 Ciudad Real, Spain; ^2^Department of Veterinary Pathobiology, Center for Veterinary Health Sciences, Oklahoma State University, Stillwater, OK 74078, USA; ^3^Centro de Biología Molecular “Severo Ochoa” (CSIC-UAM), Cantoblanco, 28049 Madrid, Spain; ^4^Utrecht Centre for Tick-borne Diseases (UCTD), Department of Infectious Diseases and Immunology, Faculty of Veterinary Medicine, Utrecht University, Yalelaan 1, 3584CL Utrecht, The Netherlands; ^5^Facultad de Medicina Veterinaria y Zootecnia, Universidad Autónoma de Tamaulipas, Km. 5 carretera Victoria-Mante, Ciudad Victoria, 87000 Tamaulipas, Mexico; ^6^Intituto Zooprofilattico Sperimentale della Sicilia, Via G. Marinuzzi no. 3, Palermo, 90129 Sicily, Italy

## Abstract

Ticks are ectoparasites of animals and humans that serve as vectors of *Anaplasma* and other pathogens that affect humans and animals worldwide. Ticks and the pathogens that they transmit have coevolved molecular interactions involving genetic traits of both the tick and the pathogen that mediate their development and survival. In this paper, the expression of heat shock proteins (HSPs) and other stress response proteins (SRPs) was characterized in ticks and cultured tick cells by proteomics and transcriptomics analyses in response to *Anaplasma* spp. infection and heat shock. The results of these studies demonstrated that the stress response was activated in ticks and cultured tick cells after *Anaplasma* spp. infection and heat shock. However, in the natural vector-pathogen relationship, HSPs and other SRPs were not strongly activated, which likely resulted from tick-pathogen coevolution. These results also demonstrated pathogen- and tick-specific differences in the expression of HSPs and other SRPs in ticks and cultured tick cells infected with *Anaplasma* spp. and suggested the existence of post-transcriptional mechanisms induced by *Anaplasma* spp. to control tick response to infection. These results illustrated the complexity of the stress response in ticks and suggested a function for the HSPs and other SRPs during *Anaplasma* spp. infection.

## 1. Introduction

Ticks are ectoparasites of wild, domestic animals and humans and are considered to be the most important arthropod vector of pathogens in some regions [1, 2]. The genus *Anaplasma* includes intraerythrocytic pathogens of ruminants, *A. marginale*, *A. centrale*, *A. bovis,* and *A. ovis* [2, 3]. Also included in this genus are *A. phagocytophilum*, which infects granulocytic leukocytes of a wide range of hosts including humans, wild and domestic animals, and *A. platys *that infects dog platelets [2, 3]. Ticks are biological vectors of *Anaplasma *spp. but different tick species transmit *A. marginale*, *A. centrale*, *A. bovis*, *A. ovis*, *A. phagocytophilum,* and *A. platys *[1]. Mammalian or tick hosts with persistent infection serve as reservoirs of these pathogens in nature [3]. 

The ticks and the pathogens that they transmit have coevolved molecular interactions involving genetic traits of both the tick and the pathogen that mediate their development and survival, but these mechanisms are not well defined [3–5]. Furthermore, although advances in proteomics technologies have been made during the last decades, proteomics studies to characterize protein expression in ticks are difficult to conduct [5–17]. Most of these studies have focused on the *sialome* (salivary gland secretory proteome) analysis of ticks [6–12] and the analysis of host-tick-pathogen interactions in an attempt to identify potential candidates for vaccine development against tick-borne diseases [5, 13–17].

The heat shock and other stress responses are a conserved reaction of cells and organisms to elevated temperatures and other stress conditions such as toxicity and pathogen infection [18–21]. The heat-shock proteins (HSPs) and other stress response proteins (SRPs) protect cells and organisms from damage, allow resumption of normal cellular and physiological activities, and overall provide higher levels tolerance to environmental stress. Crucial to cell survival is the sensitivity of proteins and enzymes to heat inactivation and denaturation. Therefore, adaptive mechanisms exist that protect cells from the proteotoxic effects of heat stress. 

At the molecular level, the heat-shock response is a transient reprogramming of cellular activities mediated by the synthesis of HSPs [18–21]. In most organisms, the major groups of HSPs, HSP100, HSP90, HSP70, HSP60, and small HSPs are represented by a few members of each class [18, 21]. HSPs are functionally linked to the large and diverse group of molecular chaperones that are defined by their capacity to recognize and bind substrate proteins that are in an unstable inactive state [18, 21]. Additionally, extracellular and membrane bound HSPs such as HSP70 are involved in binding to antigens and presenting them to the immune system [18, 20, 21].

The expression of the heat-shock genes encoding the different HSPs is primarily regulated at the transcriptional level [20]. The thermoinducibility is attributed to conserved *cis-*regulatory promoter elements (HSEs) located in the TATA-box-proximal 5′-flanking regions of heat-shock genes. The occurrence of multiple HSEs within a few hundred base pairs is a signature of most eukaryotic heat-shock genes. The eukaryotic HSE consensus sequence has been ultimately defined as alternating units of 5-nGAAn-3 [20]. HSEs are the binding sites for the *trans*active heat shock factor (HSF), and efficient binding requires at least three units, resulting in 5′-nGAAnnTTCnnGAAn-3 [20]. 

In this paper, the expression of HSPs and other SRPs was characterized in ticks and cultured tick cells by proteomics and transcriptomics analyses in response to *Anaplasma* spp. infection and heat shock. The transcriptomics analyses of ticks and tick cells in response to *A. marginale* and *A. phagocytophilum* infection and the proteomics analysis of tick cells *A. marginale* interactions were published previously [4, 5]. The proteomics analysis of tick cells *A. phagocytophilum* interactions, the proteomics and transcriptomics analyses of *R. turanicus*-*A. ovis* interactions, and the characterization of HSPs mRNAs en tick cells cultured at different temperatures are unpublished and thus methods were described here in detail. However, herein we did not present all results from these analyses, but focused on the analysis of HSPs and other SRPs expression. These results illustrated the complexity of the stress response in ticks and suggested a function for the HSPs and other SRPs during *Anaplasma* spp. infection in these organisms.

## 2. Materials and Methods

### 2.1. Ticks, Tick Cell Cultures, and Samples Preparation

ISE6 and IDE8 cells, originally derived from *Ixodes scapularis* embryos (provided by U.G. Munderloh, University of Minnesota, USA), were cultured in L15B medium as described previously [22], but for ISE6 cells the osmotic pressure was lowered by the addition of one fourth sterile water by volume. The ISE6 cells were inoculated with *A. phagocytophilum*-(NY18 isolate-) infected HL-60 cells as described previously [23, 24]. The IDE8 tick cells were inoculated with the Virginia isolate of *A. marginale* and monitored by stained smears and with phase contrast microscopy [22]. Uninfected and infected cultures (*N* = 3–5 independent cultures each) were sampled at 13 days postinfection (dpi) (percent infected cells, 26%–31%) for *A. phagocytophilum* and at 3 dpi (percent infected cells, 30%–40%) for *A. marginale*. The cells were centrifuged at 10,000 × g for 3 min and cell pellets were frozen in liquid nitrogen until used for RNA and protein extraction. Approximately 10^6^–10^7^ cells were pooled from each condition. For proteomics analysis, *A. phagocytophilum*-infected and uninfected ISE6 cells were lysed in 350 *μ*l lysis buffer (PBS, 1% Triton X-100, 1 mM sodium vanadate, 1 mM NaF, 1 mM PMSF, 1 *μ*g/ml leupeptin, and 1 *μ*g/ml pepstatin) for 30 min at 4°C. *A. marginale*-infected and uninfected IDE8 cells were lysed in 30 mM Tris-HCl, pH8.8, 7 M Urea, 2 M thiourea, and 4% CHAPS electrophoresis reagent (Sigma, St. Louis, MO, USA). Total cell extracts were centrifuged at 200 × g for 5 min to remove cellular debris. The supernatants were collected and protein concentration was determined using the Bradford Protein Assay (Bio-Rad, Hercules, CA, USA) with BSA as standard.

For analysis of HSP70 and HSP20 mRNA levels in response to heat shock, ISE6 cells were cultured in L15B medium as described previously [22] and incubated for 24 h at 4°C, 31°C (normal growth conditions), and 37°C prior to RNA extraction. Total RNA was extracted from two independent cultures for each condition as described previously [4, 5]. 

*Dermacentor variabilis, Dermacentor andersoni, *and* Rhipicephalus sanguineus *male ticks were obtained from laboratory colonies maintained at the Oklahoma State University, Tick Rearing Facility. Offhost ticks were maintained at 95% relative humidity in a 12 hr light : 12 hr dark photoperiod at 22–25°C. In order to obtain infected ticks, male ticks were fed for one week on a splenectomized calf with ascending *A. marginale* parasitemia that was experimentally infected with the Virginia isolate of *A. marginale *[5]. The ticks were then removed and maintained offhost for 4 days and then fed for an additional week on an uninfected calf to cause infection of salivary glands and other tick tissues. Uninfected ticks were fed in a similar way on the uninfected calf. *Rhipicephalus (Boophilus) microplus* male ticks (Mozambique strain) were reared in cattle at the Utrecht Centre for Tick-borne Diseases, Utrecht University, The Netherlands. Ticks were infected by feeding on a calf experimentally infected with a Texas isolate of *A. marginale *[5]. *Rhipicephalus (Boophilus) annulatus* larvae were allowed to feed on a calf naturally infected with *A. marginale* in Tamaulipas, Mexico (approximately 4% rickettsemia during tick feeding) and collected as adults after 21 days of feeding. Uninfected ticks were fed in a similar manner on an uninfected calf. The *I. scapularis* nymphs uninfected and infected with *A. phagocytophilum* (Gaillard and Dawson strains) were obtained from a laboratory colony reared at the Centers for Disease Control and Prevention, Atlanta, GA, USA [4]. Tick larvae were fed on infected or uninfected mice, collected after feeding and allowed to molt to the nymphal stage [4]. Animals were housed with the approval and supervision of the Institutional Animal Care and Use Committees. Total RNA was extracted with TriReagent (Sigma) as described previously [4, 5].

*Rhipicephalus turanicus* adult female ticks feeding on sheep were collected in farms in Sicily, Italy. DNA (for characterization of pathogen infection), RNA (for transcriptomics analysis), and proteins (for proteomics analysis) were extracted with TriReagent (Sigma) following manufacturers recommendations. *Anaplasma* spp. infection was characterized by PCR and sequence analysis of cloned major surface protein 4 (*msp4*) amplicons [25, 26]. After analysis, two ticks were positive for *A. ovis *and negative for other *Anaplasma*, *Ehrlichia, Rickettsia,* and *Theileria* spp. [26] Uninfected ticks were negative for all pathogens analyzed and were used as controls (*N* = 10) for proteomics and transcriptomics analyses. Interfering components for 2D DIGE experiments were removed from protein samples by using a 2D Clean up Kit (GE Healthcare, Madrid, Spain) according to the manufacturer's instructions. The protein pellet was resuspended in 25 *μ*l of lysis buffer (7 M urea, 2 M thiourea, 4% CHAPS, 25 mM Tris-HCl, and pH 8.0) and protein concentration was determined using the 2D Quant Kit (GE Healthcare). In order to reduce individual-to-individual variation and to obtain enough protein for analysis, protein samples that were pooled resulted in 18.5 *μ*g total protein from the infected ticks.

### 2.2. Proteomics Analysis of Tick Cells Infected with *A. phagocytophilum*

#### 2.2.1. Protein One-Step In-Gel Digestion, Peptide iTRAQ Labeling, and IEF Fractionation

Hundred *μ*g of protein extracts from each experimental condition were resuspended in a volume up to 300 *μ*l of sample buffer and applied using a 5-well comb on a conventional SDS-PAGE gel (1.5 mm-thick, 4% stacking, and 10% resolving). The run was stopped as soon as the front entered 3 mm into the resolving gel, so that the whole proteome became concentrated in the stacking/resolving gel interface. The unseparated protein bands were visualized by Coomassie Brilliant Blue R-250 staining, excised, cut into cubes (2 × 2 mm), and digested overnight at 37°C with 60 ng/*μ*l trypsin (Promega, Madison, WI, USA) at 5 : 1 protein:trypsin (w/w) ratio in 50 mM ammonium bicarbonate, pH 8.8 containing 10% (v/v) ACN, and 0.01% (w/v) 5-cyclohexyl-1-pentyl-*β*-D-maltoside (CYMAL-5) [27, 28]. The resulting tryptic peptides from each proteome were extracted by 1h-incubation in 12 mM ammonium bicarbonate, pH 8.8. TFA was added to a final concentration of 1% and the peptides were finally desalted onto C18 OASIS HLB Extraction cartridges (Waters, Milford, Massachusetts, USA) to remove the amine-containing buffers and drieddown. Dried peptides were resuspended in triethylammoniumbicarbonate (TEAB), pH 8.53 and labeled with iTRAQ reagents (Applied Biosystems, Madrid, Spain) for 1 h at room temperature. Samples from uninfected and infected ISE6 cell cultures were labeled with 116 and 117 iTRAQ tags, respectively. The two labeled samples were resuspended in 100 *μ*l 0.1% formic acid and combined into one tube. The mixture was dried down, redissolved in 3.3 ml 5 mM ammonium formiate, pH 3, cleaned up with SCX Oasis cartridges (Waters) using as elution solution 1 M ammonium formiate pH 3, containing 25% ACN, and dried down. The peptide pools were resuspended in 0.5 ml 0.1% TFA, desalted onto C18 Oasis cartridges using as elution solution 50% ACN in 5 mM ammonium formiate, pH 3 and dried down. 

The sample was taken up in focusing buffer (5% glycerol and 2% IPG buffer pH 3–10 (GE Healthcare, Madrid, Spain) loaded onto 24 wells over a 24 cm-long Immobiline DryStrip, pH3-10 (GE Healthcare), and separated by IEF on a 3100 OFFgel fractionator (Agilent, Santa Clara, CA, USA), using the standard method for peptides recommended by the manufacturer. The recovered fractions were acidified with 20 *μ*l of 1 M ammonium formiate, pH 3, and the peptides were desalted using OMIX C18 tips (Varian, Palo Alto, CA, USA). After elution with 50% ACN in 5 mM ammonium formiate, pH 3, the peptides were drieddown prior to RP-HPLC-LIT analysis.

#### 2.2.2. LC-MS/MS Analysis and Peptide Identification

All samples were analyzed by LC-MS/MS using a Surveyor LC system coupled to a linear ion trap mass spectrometer model LTQ (Thermo-Finnigan, San Jose, CA, USA) as described previously [29, 30]. The LTQ was programmed to perform a data-dependent MS/MS scan on the 15 most intense precursors detected in a full scan from 400 to 1600 amu (3 *μ* scans, 200 ms injection time, and 10 000 ions target). Singly charged ions were excluded from the MS/MS analysis. Dynamic exclusion was enabled using the following parameters: 2 repeat counts, 90 s repeat duration, 500 exclusion size list, 120 s exclusion duration, and 2.1 amu exclusion mass width. PQD parameters were set at 100 ms injection time, 8 microscans per scan, 2 amu isolation width, 28% normalized collision energy, 0.6 activation Q, and 0.3 ms activation time. For PQD spectra generation 10 000 ions were accumulated as target and automatic gain control was used to prevent overfilling of the ion trap.

Protein identification was carried out as described previously [29] using SEQUEST algorithm (Bioworks 3.2 package, Thermo Finnigan), allowing optional (Methionine oxidation) and fixed modifications (Cysteine carboxamidomethylation, Lysine, and N-terminal modification of +144.1020 Da). The MS/MS raw files were searched against the alphaproteobacteria combined with the arachnida Swissprot database (Uniprot release 15.5, 7 July, 2009) supplemented with porcine trypsin and human keratins. This joint database contains 638408 protein sequences. The same collections of MS/MS spectra were also searched against inverted databases constructed from the same target databases. The alphaproteobacteria Swissprot database was used to identify and discard *Anaplasma* and possible bacterial symbiotic sequences from further analyses. Statistical analysis and determination of error rates were performed with the Probability Ratio Method [31]. False Discovery Rate (FDR) was used as a measure of statistical significance of peptide identification and was calculated using the refined method proposed by Navarro and Vazquez [32].

#### 2.2.3. Peptide Quantification and Statistics

The intensity of the centroided iTRAQ reporter ion signals was computed by the QuiXoT software, correcting for isotope overlap between iTRAQ reporter ions [33]. The sensitivity threshold and mass tolerance for extracting the iTRAQ ratios were set to 0 and ±0.4 Da, respectively. Statistical analysis of the data was done on the basis of a novel random-effects model recently developed and validated in our laboratory that includes four different sources of variance: at the spectrum fitting, scan, peptide, and protein levels [34]. The log_2_ ratio of peptide concentration in samples *A* and *B* determined by scan *s* coming from peptide *p* derived from protein *q* is expressed as *x*_qps_ = log _2_ (*A*/*B*). The statistical weight associated to the scan, *w*_qps_, is calculated from the spectrum fitting and the scan variance, *σ*_**s**_^2^, as described in [34]. The log_2_-ratio value associated to each peptide, *x*_qp_, is calculated as a weighted average of the scans used to quantify the peptide, and the value associated to each protein, *x*_q_, is similarly the weighted average of its peptides. Besides, a grand mean, *x*, is calculated as a weighted average of the protein values. In turn, the statistical weight associated to each peptide, *w*_qp_, is calculated from the corresponding scan weights and the peptide variance, *σ*_**P**_^2^, and that of each protein, *w*_**q**_, is calculated from the corresponding peptide weights and the protein variance, *σ*_**Q**_^2^. In all cases, the statistical weights are the inverses of variances. Outliers at the scan and peptide levels are detected by calculating the probability that the measurements deviate from the expected average according to their respective variances, and controlling for the false discovery rate at each level, FDR_qps_ and FDR_qp_, respectively. Details about the statistical model and the algorithm used to calculate the variances at the scan, peptide, and protein levels can be found in Jorge et al. [34]. Differential protein expression in early versus late infections was compared using Venn diagrams to show shared and distinct protein expression. Significance of overlaps was calculated using hypergeometric distributional assumption [35], and *P*-values were adjusted using Bonferroni correction for multiple comparisons [36]. Protein ontology for biological process (BP) of differentially expressed proteins was done using the human protein databases at http://www.hprd.org/ and http://www.ebi.ac.uk/interpro/. The proportion of up- and downrepresented proteins was statistically analyzed separately for early and late infections for each BP protein ontology category by a Fisher two-tailed test (*P* = .05) using Statistica 6.0 software (StatSoft Inc.,12, OK, USA).

### 2.3. Proteomics Analysis of Tick Cells Infected with *A. marginale*

Proteomics analysis was performed at Applied Biomics (Hayward, CA, USA; http://www.appliedbiomics.com/) by two-dimensional difference in gel electrophoresis (2D DIGE) as reported previously [5].

### 2.4. Proteomics Analysis of Ticks Naturally Infected with *A. ovis*

#### 2.4.1. Two-Dimensional Difference in Gel Electrophoresis (2D DIGE)

CyDye DIGE fluor labeling kit for scarce protein samples (GE Healthcare) was used to label tick proteins according to the manufacturer's protocol. Briefly, for cysteine reduction before labeling, 5 *μ*g of protein of each sample were incubated with 2 nmol Tris (2carboxyethyl) phosphine hydrochloride (TCEP; Sigma) at 37°C for 1 hour in the dark and, for labeling, 4 nmol of Cy5 dye in 2 *μ*l of anhydrous DMF (Sigma) were added and the samples were incubated at 37°C for 30 min in the dark. For internal standardization, a pool of equal amounts of all samples (5 *μ*g per sample) was created and labeled with Cy3 dye with the same procedure but scaling adjusted the quantities of reagents according to the amount of protein (10 *μ*g). The reaction was quenched by adding an equal volume of 2 x sample buffer (7 M urea, 2 M thiourea, 4% w/v CHAPS, 1% v/v IPG buffer pH 3–11, and 0.2% w/v DTT). Before 2D separation, 5 *μ*g of the Cy3-pool was mixed with 5 *μ*g of each sample. 

For the first dimension, 24-cm 3–11 NL pH range IPG strips were rehydrated overnight in 450 *μ*L of DeStreak Rehydration Solution (GE Healthcare) supplemented with 0.5% IPG buffer pH 3–11 (GE Healthcare) using a reswelling tray. IEF was performed at 20°C using an Ettan IPGphor 3 (GE Healthcare). Samples were applied using anodic cup loading and the isoelectrofocusing was carried out using the following conditions: 300 V for 3 h, 300–1000 V for 6 h, 1000–10000 V for 3 h, 10000 V for 3 h, and 500 V for 3 h. Prior to second dimension, focused IPG strips were incubated for 10 min equilibration buffer containing 50 mM Tris-HCl pH 8.8, 6 M urea, 30% v/v glycerol, 2% w/v SDS, 0.5% w/v DTT, and traces of bromophenol blue. Equilibrated IPG strips were placed onto 12% homogeneous SDS-polyacrylamide gels casted in low-fluorescence glass plates using an Ettan-DALT Six System (GE Healthcare). Electrophoresis was carried out at 20°C and 0.5 W/gel for 30 min followed by a second step at 15 W/gel for 4 hours.

#### 2.4.2. Image Acquisition and Data Analysis

Proteins were visualized using an Ettan DIGE Imager (GE Healthcare) following the manufacturer's instructions. Image analysis was performed with DeCyder 2D Software, version 7.0 (GE Healthcare). Four images were considered for the analysis, 2 corresponded to samples labeled with Cy5, and 2 corresponded to the sample pool labeled with Cy3 and acquired individually with each gel. Spot codetection, normalization of each spot against the corresponding value of the internal pool, and volume ratios calculation were carried out using Differential In-Gel Analysis (DIA) module. In the Biological Variation Analysis (BVA) module, the 4 spot maps were distributed in 3 groups, that is, standard, and the 2 different samples (one control and one infected) and the standard image most representative with average quality were assigned as master. After match images, average ratios between groups were calculated. Protein spots with 5-fold as threshold in the average ratio were considered as differentially expressed between samples under comparison.

#### 2.4.3. Selection and Preparation of Protein Samples for Mass Spectrometry

For preparative gel, equal protein amounts of all samples were pooled. 2D electrophoresis was carried out in the same conditions described above for CyDye labeled samples, but in this case, after second dimension, the gel was stained with Sypro Ruby (Molecular Probes, Invitrogen, Eugene, OR, USA) following the protocol recommended by the manufacturer. Proteins were visualized by fluorescence using an Ettan DIGE Imager (GE Healthcare) selecting 100 *μ*m as pixel size and channel Sypro Ruby 1 with 0.4 of exposure and gel image was acquired. The gel was matched automatically in the BVA module of DeCyder software with the DIGE image in order to select the spots of interest for mass spectrometry analysis. The 2D electrophoresis stained gel was washed twice for 10 min with distilled water. Selected protein spots were visualized with a UV benchtop transilluminator (UVP, Cambridge, UK), manually excised from the gels, dehydrated with acetonitrile, and vacuum dried (Savant Speed Vac, mod SPD, 121 P, equipped with a vacuum pump OFP-400). After drying, spots were rehydrated and digested *in situ* with trypsin (Promega) as described by Shevchenko et al. [27] with minor modifications. Stained protein gel spots were incubated in 50 mM NH_4_HCO_3_ with trypsin (5 ng/*μ*l) for 1 hr in an ice bath. The digestion buffer was removed and gels were covered again with 50 mM NH_4_HCO_3_ and incubated at 37°C for 12 hr. Whole supernatants were allowed to dry and then stored at 20°C until mass spectrometry analysis.

#### 2.4.4. Matrix-Assisted Laser Desorption/Ionization Time of Flight Mass Spectrometry (MALDI-TOF MS) Analysis

Peptide mass fingerprinting was conducted as described previously [37] using an Autoflex (Bruker Daltonics, Bremen, Germany) mass spectrometer in a positive ion reflector mode employing 2, 5-dihydroxybenzoic acid as matrix, and an AnchorChip surface target (Bruker Daltonics). Peak identification and monoisotopic peptide mass assignation were performed automatically using Flexanalysis software, version 2.2 (Bruker Daltonics). Database searches were performed using MASCOT (http://matrixscience.com/) [38] against the NCBI nonredundant protein sequence database (http://www.ncbi.nih.gov/). The selected search parameters were as follows: tolerance of two missed cleavages, carbamidomethylation (Cys), and oxidation (Met) as fixed and variable modifications, respectively, and setting peptide tolerance to 100 ppm after close-external calibration. A significant MASCOT probability score (*P* < .05) was considered as condition for successful protein identification.

#### 2.4.5. Reverse Phase-Liquid Chromatography (RP-LC) MS/MS Analysis

When peptide mass fingerprinting failed to identify a spot, the protein digest was dried, resuspended in 7 *μ*l of 0.1% formic acid, and analyzed by RP-LC-MS/MS in a Surveyor HPLC system coupled to an ion trap Deca XP mass spectrometer (Thermo Fisher Scientific, Waltham, MA, USA). The peptides were separated by reverse phase chromatography using a 0.18 mm × 150 mm BioBasic C18 RP column (Thermo Fisher Scientific), operating at 1.8 *μ*l/min. Peptides were eluted using a 50-min gradient from 5 to 40% solvent B (Solvent A: 0,1% formic acid in water, solvent B: 0,1% formic acid, 80 % acetonitrile in water). ESI ionization was done using a microspray “metal needle kit” (Thermo Fisher Scientific) interface. Peptides were detected in survey scans from 400 to 1600 amu (8 *μ*scans), followed by three data-dependent MS/MS scans, using an isolation width of 3 amu, normalized collision energy of 30%, and dynamic exclusion, applied during 3-min periods. Peptide identification from raw data was carried out using the SEQUEST algorithm (Bioworks Browser 3.2, Thermo Fisher Scientific) and the PHENYX 2.6 search engine (GENEBIO, Switzerland). Database search was performed against the Apicomplexa, *α*proteobacteria, and metazoa databases download from the Protein Knowledgebase (UniProtKB) (http://www.uniprot.org/). The following constraints were used for the searches: tryptic cleavage after Arg and Lys, up to two missed cleavage sites, and tolerances of 2 Da for precursor ions, and 0.8 Da for MS/MS fragment ions and the searches were performed allowing optional Met oxidation and fixed Cys carbamidomethylation. If the Sequest and Phenyx searches did not yield positive results, high-quality spectra that had not been assigned to any protein identification were selected and a manual *de novo* interpretation was conducted that was confirmed with PEAKS Studio 4.5 software (Bioinformatics Solutions Inc., Waterloo, ON, Canada).

### 2.5. Real-Time RT-PCR

Real-time RT-PCR was performed on RNA samples from IDE8 and ISE6 tick cells and ticks with gene-specific primers ([4, 5] and HSP20F: 5′-GACAACTGCGTCGTAGTCCA-3′ and HSP20R: 5′-CTTGACAGCACCTCCTTTGG-3′, and HSP70F: 5′-GTTTTCAAGAATGGGCGTGT-3′ and HSP70R: 5′-GAGGCTTGCTGTTCTTGTCC-3′ for HSP20 and HSP70, resp.) using the iScript One-Step RT-PCR Kit with SYBR Green and the iQ5 thermal cycler (Bio-Rad, Hercules, CA, USA) following manufacturer's recommendations. A dissociation curve was run at the end of the reaction to ensure that only one amplicon was formed and that the amplicon denatured consistently in the same temperature range for every sample [39]. The mRNA levels were normalized against tick 16S rRNA [4, 5] using the genNorm method (ddCT method as implemented by Bio-Rad iQ5 Standard Edition, Version 2.0) [40]. Data from cells cultured at 31°C (normal growth conditions) were compared with data from cells cultured at 4°C and 37°C and between *Anaplasma*-infected and uninfected ticks and tick cells using the Student's *t*-test (*P* = .05).

## 3. Results and Discussion

**Figure 1:**
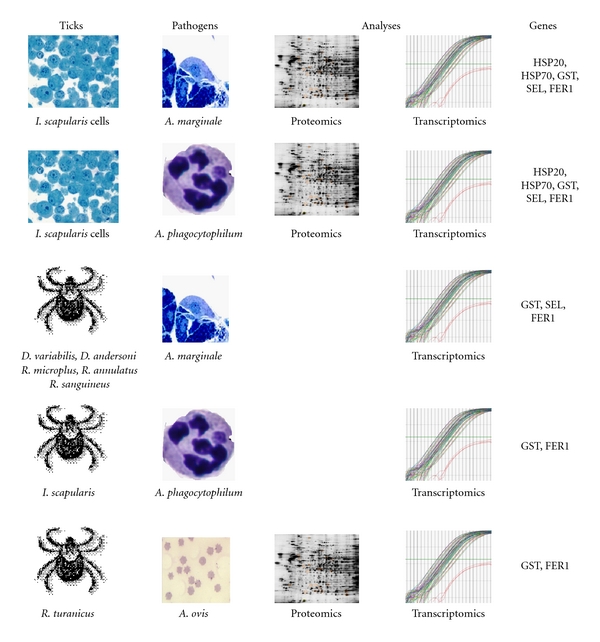
Experimental design for the analysis of heat shock and other stress response genes/proteins in ticks and cultured tick cells in response to *Anaplasma* spp. infection.

In this study, proteomics and transcriptomics were used to characterize heat shock and other stress responses in ticks and tick cells in response to *Anaplasma* spp. infection and heat shock (Figure 1). These analyses included both natural (*I. scapularis* ticks and cells *A. phagocytophilum*, *D. variabilis/D. andersoni/R. microplus/R. annulatus/R. sanguineus*-*A. marginale*, and *R. turanicus*-*A. ovis*) and nonnatural (*I. scapularis* cells *A. marginale*) tick-pathogen interactions (Figure 1). Gene/proteins considered in this paper included HSPs and other SRPs such as glutathione-S transferase (GST), selenoprotein (SEL), metallothionein (MET), and ferritin1 (FER1). These proteins have been shown to be involved in the cellular response to different stress conditions such as heat shock (HSPs; [18, 19, 21]), endogenous and environmental chemicals (GST; [41]), oxidative stress (SEL, MET, FER1; [42–44]), and metals (MET, FER1; [43, 44]). Additionally, these proteins have been reported to be regulated by tick attachment, blood feeding, or pathogen infection [4, 5, 14, 15, 45–51] as well as expressed in unfed and uninfected ticks and tick cells [10, 11, 52, 53].

### 3.1. Analysis of HSPs and Other SRPs in Cultured *I. scapularis* Tick Cells in Response to *A. marginale* Infection

**Figure 2:**
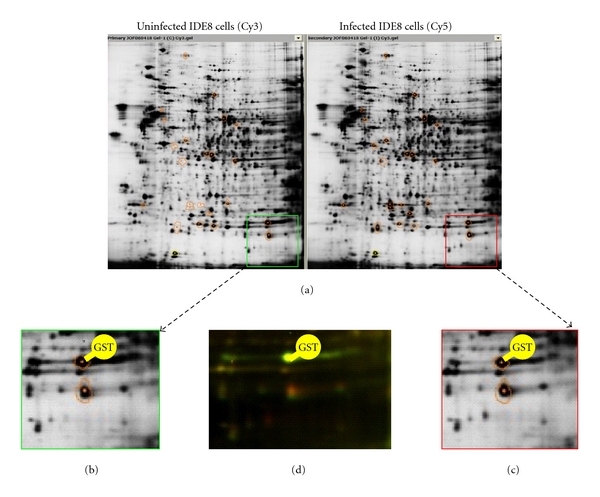
2D DIGE analysis of the total proteome of uninfected and* A. marginale*-infected *I. scapularis* IDE8 tick cells showing GST underexpression in infected cells [5]. (a) 2D representative maps of uninfected (left) and infected (right) IDE8 cells that were labeled with Cy3 and Cy5, respectively. Circled spots correspond to proteins that were differentially expressed after infection. (b, c) Amplification of gel area where GST protein was localized after MS identification in uninfected (green square) and infected (red square) IDE8 cells. (d) 2D DIGE gel image corresponding to the overlapping Cy3 and Cy5 fluorochromes for uninfected versus infected paired samples in the GST region mentioned above.

At the mRNA level, HSP70, GST, SEL W2a, and salivary SEL M genes were upregulated while FER1 was downregulated in *A. marginale*-infected IDE8 tick cells [4, 5]. The mRNA levels for HSP20 and HSP70 were further evaluated by real-time RT-PCR in ISE6 tick cells in response to *A. marginale* infection. The results showed that both HSP20 (2.6 ± 2.4 infected to uninfected cells mRNA ratio, Ave ± SD) and HSP70 (2.4 ± 1.2) were upregulated in *A. marginale*-infected tick cells. These results suggested that the stress response was activated in cultured *I. scapularis* tick cells in response to *A. marginale *infection [5]. However, at the protein level, GST was underexpressed in infected IDE8 tick cells [5], probably reflecting a posttranscriptional mechanism induced by *A. marginale* to control tick stress response to infection (Figure 2). In fact, functional analyses conducted by RNA interference (RNAi) in IDE8 tick cells demonstrated that GST gene knockdown resulted in lower *A. marginale* infection levels, thus suggesting that while GST gene expression is activated in response to pathogen infection, it is required for *A. marginale* infection, trafficking, and/or multiplication in tick cells [5, 54].

### 3.2. Analysis of HSPs and Other SRPs in Cultured *I. scapularis* Tick Cells in Response to *A. phagocytophilum* Infection

Proteomics analysis of ISE6 tick cells in response to *A. phagocytophilum* infection demonstrated that while HSP70 was overexpressed in infected cells, other putative HSPs such as HSP20 were underexpressed after infection (Table 1). However, HSPs represented only 10% (3/31) and 4% (2/50) of over and underexpressed proteins, respectively, in *A. phagocytophilum*-infected ISE6 tick cells (unpublished results). At the mRNA level, FER1, SEL W2a, SEL M, and GST expression did not change significantly between *A. phagocytophilum*-infected and uninfected ISE6 tick cells [4]. The mRNA levels for HSP20 and HSP70 were evaluated by real-time RT-PCR in ISE6 tick cells in response to *A. phagocytophilum* infection. As with other SRPs, the results showed that both HSP20 (8 ± 8 infected to uninfected cells mRNA ratio, Ave ± SD) and HSP70 (0.5 ± 0.4) mRNA levels did not change significantly in *A. phagocytophilum*-infected ISE6 tick cells when compared to uninfected cells. These results demonstrated differences in the response of ISE6 tick cells to *A. marginale* and *A. phagocytophilum* infections [4]. As discussed previously for GST protein expression in *A. marginale*-infected IDE8 tick cells, differences in HSP expression between proteomics and transcriptomics analyses probably reflected a posttranscriptional mechanism induced by *A. phagocytophilum* to control tick responses to infection.

### 3.3. Analysis of HSPs and Other SRPs in Anaplasma-Infected Ticks

In *A. marginale*-infected *D. variabilis* male ticks, FER1 mRNA levels were lower in the guts and did not change in the salivary glands of infected ticks [5]. For GST, mRNA levels did not change in both guts and salivary glands after infection [5]. In *A. marginale*-infected *D. andersoni* male ticks, FER1 mRNA levels did not change in guts and salivary glands, and GST mRNA levels were similar and lower in guts and salivary glands, respectively, when compared to uninfected controls. While SEL M and FER1 were not differentially expressed in *R. microplus* salivary glands, GST mRNA levels were significantly higher in uninfected ticks. In *R. annulatus* and *R. sanguineus*, *A. marginale* infection did not change GST and FER1 mRNA levels in guts and salivary glands. These results demonstrated differences between tick species in the stress response to *A. marginale* infection. 

In *A. phagocytophilum*-infected *I. scapularis* nymphs, the expressions of FER1 and GST were significantly downregulated at the mRNA level [4]. The mRNA levels were similar in *I. scapularis* nymphs infected with two different strains of *A. phagocytophilum *[4]. However, as shown before in ISE6 tick cells, these results were different from those obtained in response to *A. marginale* infection and may reflect pathogen-specific and/or tick species-specific differences in the effect of *Anaplasma *spp. infection on gene expression. 

Proteomics analysis of *R. turanicus* ticks infected with *A. ovis* was conducted in comparison with their respective uninfected controls and the proteins that were differentially expressed with an average ratio of ±5-fold after DeCyder software analysis of DIGE gels was considered. Two experiments were conducted with similar results. Of the 50 identified differentially expressed proteins (30 overexpressed and 20 underexpressed in infected ticks), none corresponded to HSPs or other SRPs. At the mRNA level, GST was downregulated (0.008 ± 0.007 infected to uninfected cells mRNA ratio, Ave ± SD) while FER1 expression (0.02 ± 0.02) did not change in infected ticks. 

**Figure 3:**
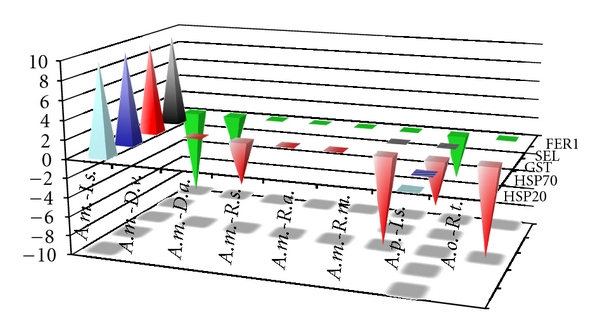
Differential expression of heat shock and other stress response genes in different tick-*Anaplasma* interactions. The mRNA levels of HSP20, HSP70, GST, SEL, and FER1 were characterized by real-time RT-PCR in uninfected and *Anaplasma*-infected ticks and tick cells. Arbitrarily, +10 and −10 values were used to represent gene upregulation and downregulation, respectively. When the mRNA levels did not change after pathogen infection, a zero value was used. A ±5 value was used when pathogen infection was characterized in the same species ticks and tick cells or in tick guts and salivary glands. Abbreviations: *A. m.*, *A. marginale*; *A. p.*, *A. phagocytophilum*; *A. o.*, *A. ovis*; *I.s.*, *I. scapularis*; *D. v.*, *D. variabilis*; *D. a.*, *D. andersoni*; *R. s.*, *R. sanguineus*; *R. a.*, *R. annulatus*; *R. m.*, *R. microplus*; *R. t.*, *R. turanicus*.

*R. turanicus* is a natural vector of *A. ovis* [55]. *D. variabilis*, *D. andersoni*, *R. microplus*, *R. annulatus,* and *R. sanguineus* are natural vectors of *A. marginale,* and *I. scapularis* is a natural vector of *A. phagocytophilum* in different regions of the world [1–3]. However, *I. scapularis* does not vector *A. marginale* [1–3]. The results of HSPs and other SRPs expression suggested that, at least when ticks are the pathogen's natural vector, heat shock and other stress responses are not strongly activated, probably reflecting tick-pathogen coevolution [3–5]. This fact was demonstrated in *A. marginale*-infected *D. variabilis*, *D. andersoni*, *R. microplus*, *R. annulatus,* and *R. sanguineus*, in *A. phagocytophilum*-infected *I. scapularis* ticks and ISE6 tick cells, and in *A. ovis*-infected *R. turanicus*, but not in *A. marginale*-infected IDE8-cultured tick cells, which were not derived from a natural vector species [1–3] (Figure 3). In fact, except for FER1 expression that was consistently downregulated or did not change in response to *Anaplasma* infection, the expression of the other stress response genes was upregulated in *A. marginale*-infected *I. scapularis* IDE8 cells while did not change or were downregulated in other tick-*Anaplasma* interactions (Figure 3). These results suggested that while cultured tick cells are a useful tool for the study of tick-*Anaplasma* interactions, experiments should be conducted with the natural tick vector and pathogen.

### 3.4. Expression of HSP20 and HSP70 Genes in *I. scapularis* ISE6 Tick Cells in Response to Heat Shock

**Figure 4:**
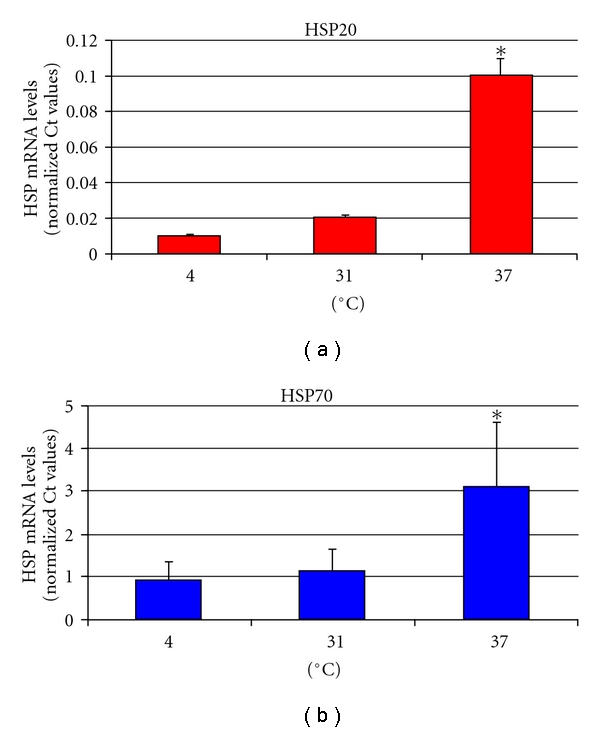
Heat shock response in *I. scapularis* ISE6 tick cells. ISE6 cells were incubated for 24 h at 4°C, 31°C (normal growth conditions), and 37°C prior to RNA extraction. Total RNA was extracted from two independent cultures for each condition, and HSP20 and HSP70 mRNA levels were analyzed by real-time RT-PCR. The mRNA levels were normalized against tick 16S rRNA and compared between control cells grown at 31°C and cells cultured at 4°C and 37°C using the Student's *t*-test (*P < .05).

To further characterize the expression of HSP20 and HSP70 genes in response to heat shock, the mRNA levels for HSP20 and HSP70 were evaluated by real-time RT-PCR in ISE6 tick cells incubated at different temperatures. The results demonstrated that both HSP20 and HSP70 were upregulated after heat shock at 37°C but not at 4°C when compared to control cells grown at 31°C (Figure 4). Although the mRNA levels for HSP70 were 72-fold higher than those for HSP20 at 31°C, a 5- and 3-fold increase in HSP20 and HSP70 mRNA levels was obtained after heat shock at 37°C, respectively, (Figure 4). These results showed that, as in other organisms, HSPs are upregulated in tick cells in response to heat shock.

## 4. Conclusions

The results of these studies demonstrated that the stress response was activated in ticks and cultured tick cells after *Anaplasma *spp. infection and heat shock. However, under natural vector-pathogen relationships, HSPs and other SRPs were not strongly activated, probably reflecting tick-pathogen coevolution. Nevertheless, at least as shown by proteomics analysis of ISE6 tick cells in response to *A. phagocytophilum* infection, some HSPs such as the HSP70 family were overexpressed while other putative HSPs such as HSP20 were underexpressed in infected cells. Furthermore, these results demonstrated pathogen-specific and tick species-specific differences in the expression of HSPs and other SRPs in ticks and tick cells infected with *Anaplasma* spp. Additionally, our results suggested the existence of posttranscriptional mechanisms induced by *Anaplasma* spp. to control tick response to infection. In summary, the results presented herein illustrate the complexity of the stress response in ticks and suggest a function for HSPs and other SRPs during *Anaplasma* infection in ticks.

## Figures and Tables

**Table 1:** HSP differential expression between *A. phagocytophilum*-infected and control-uninfected *I. scapularis* ISE6 tick cells.

Protein description	Fold change	UNIPROT accession number	FDR
HSP70-2	+1.42	B4YTT9	0.000
HSP70-1	+1.30	B4YTT8	0.002
HSP70	+1.20	B7PEN4	0.011
HSP, putative	−1.45	B7P1Z8	0.016
HSP20, putative	−5.81	B7P7F7	0.004

^a^+ and − indicate protein overexpression and underexpression in *A. phagocytophilum*-infected cells, respectively.

^b^False discovery rate (FDR) was used as a measure of statistical significance of peptide identification and was calculated using the refined method proposed by Navarro and Vazquez [32].
